# The relationship of psychological reactance, health locus of control and sense of self-efficacy with adherence to treatment in psychiatric outpatients with depression

**DOI:** 10.1186/s12888-014-0324-6

**Published:** 2014-11-21

**Authors:** Carlos De las Cuevas, Wenceslao Peñate, Emilio J Sanz

**Affiliations:** Professor of Psychiatry, Department of Internal Medicine, Dermatology and Psychiatry, University of La Laguna, Canary Islands, Spain; Red de Investigación en Servicios de Salud en Enfermedades Crónicas (REDISSEC), Tenerife, Spain; Professor of Psychology, Department of Personality, Assessment and Psychological Treatments, University of La Laguna, Canary Islands, Spain; Professor of Clinical Pharmacology, Department of Physical Medicine and Pharmacology, University of La Laguna, Canary Islands, Spain

**Keywords:** Adherence, Depressive disorders, Health locus of control, Psychological reactance, Self-efficacy

## Abstract

**Background:**

Although non-adherence to antidepressant medications is a significant barrier to the successful treatment of depression in clinical practice, few potentially modifiable predictors of poor adherence to antidepressant treatment are known. The aim of this study was to examine the relationship of psychological reactance, health locus of control and the sense of self-efficacy on adherence to treatment regimen among psychiatric outpatients with depression.

**Methods:**

One hundred and forty-five consecutive psychiatric outpatients suffering from depressive disorders were invited to participate in a cross-sectional study and 119 accepted. Patients completed a series of self-reported questionnaires assessing psychological reactance, health locus of control, self-efficacy, and adherence to prescribed medication in addition to socio-demographic and clinical variables. Logistic regression analyses were performed to determine which factors better correlate to treatment adherence.

**Results:**

Age was found to be the best correlate of adherence to prescribed treatment. As regards psychological dimension studied, medication adherence was negatively associated with both cognitive and affective psychological reactance; patients with higher psychological reactance were more likely to be noncompliant than patients showing a low level of psychological reactance. Regarding health locus of control, only the external dimension of doctor-attributed health locus of control was positively associated with medications adherence. No effect on adherence was observed for the self-efficacy scale.

**Conclusions:**

Psychological reactance is an important correlate of adherence to treatment in patients with depressive disorders and this needs to be considered when giving clinical advice in order to avoid inducing reactance and thus non-adherence to prescribed treatments. Mental health professionals need to learn about communication techniques and counseling skills that enable them to deal with the psychological reactance of their patients.

## Background

Depressive disorders are a major problem for public health because of their very common presentation. They are a major cause of disability and can cause death both by suicide and because of increased rates of physical disorders [1]. Until two decades ago, depression was considered an acute and self-limiting episodic illness. However, long-term naturalistic studies have revealed the importance of considering depression as a chronic lifelong disease with different possible clinical courses in which symptom severity transverse judgments provide limited prognostic information. Approximately 70% of patients recover from major depressive episode during the first year [2]. However, of those patients who do not recover within the first year, 10% to 15% remain ill for at least five years [3]. Of the patients who recover from an episode of major depression, 22% relapse during the first year of monitoring [4]. Only 25% of patients experienced a single episode of depression while 75% suffer from a recurring depression, with one or more recurrences throughout life [5]. In addition, between one quarter and two-thirds of patients with major depression suffer comorbid dysthymia [6].

Non-adherence to antidepressant medications is a significant barrier to the successful treatment of depression in clinical practice, and this has been linked to poor treatment outcomes such as increased risk of relapse and recurrence, in addition to increased healthcare costs [7-9].

After four decades of compliance research, the literature shows that treatment adherence in psychiatric practice is a multifactorial issue that includes patient-centered factors, therapy-related factors, healthcare system factors, clinician-patient alliance and communication factors, social and economic factors, and disease factors [10-13]. However, despite the relevance of the contribution of that group of variables in predicting adherence, considerable accounted variance remains. A recent systematic review on compliance with prescribed treatment in patients with depressive disorders has revealed there is no evidence of a substantial relationship between compliance and socio-demographic or clinical variables [13]. Under these circumstances, there is an imperative need to identify major determinants of non-adherence, particularly those modifiable and thus amenable to intervention, which could facilitate the optimal use of psychotropic medications.

The adherence construct has been reported as a health-related habit comprised of a set of very different behaviors, which are not always related to each other [14]. The health belief model that postulates that health behaviors are expressions of health beliefs has been proposed to account for non-adherence to prescribed treatments [15]. Patients' health cognitions should contribute to medication adherence since they reflect patients' beliefs regarding the extent to which they are able to control or influence their health outcomes based on their personal experience, the course of their illnesses and the different treatments used.

Having control, or the belief of having control, over one’s health has been hypothesized to moderate personal health-promoting behavior [16,17] and has been shown to be of importance for the wellbeing, quality of life and functioning of patients with chronic psychiatric disorders [18-20]. Nevertheless, few studies are available concerning factors that may influence patients’ sense of control and that enable finding strategies in mental health interventions to promote these patients’ sense of control and adherence.

The study of psychological reactance, health locus of control and the sense of self-efficacy could be of crucial importance and can help to increase knowledge about patient’s adherence to treatment. Psychological reactance is an aversive affective reaction in response to regulations or impositions that impinge on freedom and autonomy [21,22], and by means of limiting and threatening freedom, recommendations to follow a medication have the potential to elicit reactance and, as a result, lead patients to ignore the recommended treatment. Health locus of control refers to the individual’s beliefs regarding the control of health [16]. A patient with an internal locus of control believes that health outcomes are a direct result of their own behavior, while a patient with an external locus of control believes that health outcomes are a result of either chance or other powerful people, such as physicians. Finally, sense of self-efficacy reflects the individual’s belief in their own skills to plan and perform certain activities to attain particular aims [23,24] and leads to a greater sense of confidence and control, translating into a greater theoretic likelihood of both intending to perform the behavior and actually doing so.

None of these psychological characteristics―potential determinants of adherence to treatment―have been jointly investigated in depressive disorders. Thus, the aim of this study was to examine the individual and joint effects of psychological reactance, health control beliefs, and the sense of self-efficacy on adherence to a treatment regimen among psychiatric outpatients with depression. We expected health locus of control to have a marked effect on adherence to a treatment regimen, such that internally oriented patients would adhere more than externally oriented patients. In the same way, we hypothesized that psychological reactance would also have a marked effect on adherence to a treatment regimen, such that patients with low reactance would comply with their physicians' guidelines and instructions more than patients with high reactance that would oppose them. Finally, we expected self-efficacy to have a considerable effect on adherence to a treatment regimen, such that patients with high self-efficacy would adhere more than patients with low self-efficacy.

## Methods

### Sample

From January 2013 to May 2013, one hundred and forty-five consecutive psychiatric outpatients suffering from depressive disorders seen in two Community Mental Health Centers on Tenerife Island (Canary Islands, Spain) were invited to participate in a cross-sectional study; a total of 119 accepted. Patients were eligible for inclusion in the study if they were aged 18 and over and were diagnosed by their psychiatrists with mood disorders using the International Classification of Diseases, Tenth Edition (ICD-10) codes F31 (bipolar affective disorder), F32 (depressive episode), F33 (recurrent depressive disorder), and F34.1 (Dysthymia). Inclusion into the study required patients to have received at least one drug prescription. Each participant received a full explanation of the study, after which they signed an informed consent document approved by the clinical research ethics committee of Nuestra Señora de Candelaria Teaching Hospital in Santa Cruz de Tenerife. Each participant then filled out a brief socio-demographic survey and the remaining questionnaires.

### Measures

#### Socio-demographic characteristics and clinical variables

Age, sex, educational level (no formal education, primary studies, secondary studies, and university degree), history as psychiatric patient (in years), and type of psychoactive drugs currently taken were assessed. For assessment purposes the drugs were divided into the common groups of psychotropic drugs: antidepressants (tricyclics, selective serotonin reuptake inhibitors [SSRIs] and serotonin and norepinephrine selective reuptake inhibitors [SNRIs]), benzodiazepines, antipsychotics (conventional and atypical) and mood stabilizers. For statistical analysis purposes, a new variable (number of different drugs) was drawn up as an indirect measure of treatment complexity. We also recorded how long patients had been under psychiatric treatment (in months), the number of different psychiatrists treating them during that time, and the number of psychiatric admissions specifying their voluntary or involuntary character. Psychiatrists responsible for patient mental health care were asked about patient diagnosis.

### Self-reported adherence

Self-reported adherence to psychiatric medication prescribed was assessed using the Morisky scale [25] in its Spanish correctly validated version [26], a simple four-item yes/no self-report instrument commonly used to assess patient adherence to treatment across a variety of chronic medical and psychiatric conditions, including affective disorders [27,28]. The Morisky scale includes items querying whether the patient ever forgot to take medications, was careless with medications, stopped taking medications at times when feeling better or discontinued medications when feeling worse. This scale enables classifying patients as compliant or non-compliant. Patients are classified as non-adherent when they answered “yes” to at least one of the four questions. The scale can also be used as a continuous variable, where maximum adherence is defined as answering “no” to every question. The scale has been shown to have good reliability (*α* = .83), and concurrent and predictive validity in outpatient settings [25,29].

### Clinical status

#### Beck depression inventory (BDI-II)

The Beck Depression Inventory II (BDI-II) is a 21-item self-report questionnaire that assesses cognitive, behavioral, affective, and somatic symptoms of depression, developed to correspond to the criteria for DSM-IV depressive diagnoses [30]. Each item is scored by the subject on a four-point scale (0–3) according to the way the participant has been feeling in the previous two weeks. The 21 items are then summed to give a single total score for the BDI-II, for which cutoff scores have previously been established. The cutoffs used differ from the original: 0–13: minimal depression; 14–19: mild depression; 20–28: moderate depression; and 29–63: severe depression. Higher total scores indicate more severe depression-related symptoms. The BDI-II is a reliable and well-validated measure in screening for depression symptoms in adults, with Cronbach's alphas ranging from 0.73 to 0.95. The Spanish validated version by Sanz et al. (2005) [31] was used.

### Psychological features correlates

Three questionnaires were used in the study: the Hong Psychological Reactance Scale (HPRS), Form C of the Multidimensional Health Locus of Control Scale (MHLCS) and the General Self-Efficacy Scale (GSE).

The Hong Psychological Reactance Scale [32] is a 14-item self-report questionnaire designed to measure the individual difference in reactance proneness, that is, a person's trait propensity to experience psychological reactance. Psychological Reactance [16] assumes that, when an individual's freedom is threatened, the individual will be motivated to restore the perceived loss of freedom. Participants indicated the extent to which they endorsed each cognitive or affective statement on a five-point Likert scale (1 = strongly disagree to 5 = strongly agree). The study used the validated Spanish version of the scale [33].

Form C of the Multidimensional Health Locus of Control (MHLC) Scale [34] was used to assess patients’ perception about ‘who’ controls their depression outcomes. MHLC is an 18-item, general purpose, condition-specific locus of control self-report scale that could easily be adapted for use with any medical or health-related condition to assess an individual’s belief on what influences their health. It is comprised of four subscales: one internal locus of control subscale: internality and three external locus of control scales: chance, doctors, and other (powerful) people that measure control variables in regard to participants’ health. Each item consists of a belief statement about patient medical condition with which they may agree or disagree by means of a six-point Likert scale ranging from strongly disagree [1] to strongly agree [6]. The study used the validated Spanish version of the scale [35].

The General Perceived Self-Efficacy Scale [36] is a 10-item self-report scale that measures general self-efficacy as a prospective and operative construct. In contrast to other scales designed to assess optimism, this scale explicitly refers to personal agency, i.e., the belief that one's actions are responsible for successful outcomes. Each item is scored from 1 (not at all true) to 4 (completely true). The summary score ranges from 10 to 40, with the highest score indicating high self-efficacy. The study used the validated Spanish version of the scale [37].

### Data analysis

Socio-demographic and clinical characteristics were assessed according to descriptive analyses. An initial correlation analysis was performed to assess the relationship between psychological processes and their relationship pattern with traditional contextual variables associated with adherence to treatment. To contrast adherent and non-adherent patients in the different variables, one-factor ANOVA was performed. To predict adherence a logistic regression analysis was performed to introduce both nominal and continuous variables together. In order to test differences in subscale scores, given the different number of items in the MHLC-C and Psychological Reactance subscales, each subscale score was computed as the mean of item scores. Also, in order to contrast the specific role for type of medication used, chi-squared analyses were performed. Statistical analyses were performed using the software package SPSS19.

## Results

We recorded a high response rate of 82.1% resulting in a sample of 119 psychiatric outpatients suffering from depressive disorders. Table 1 shows the sample distribution according to socio-demographic and clinical variables included in the study. According to the Morisky scale total scores, slightly more than half the patients (60, 50.4%) were classified as non-adherent to the treatment prescribed.

**Table 1:** **Socio-demographic and clinical characteristics of the sample studied**

**Variable**	**Category**	**Number of cases**	**% of the sample**
**Age**	30-45 years	26	21.8
*Mean Age 56.1 ± 12.0*	45-60 years	53	44.5
*Rank 30-82*	60-75 years	31	26.1
	> 75 years	9	7.6
**Sex**	Male	28	23.5
	Female	91	76.5
**Educational level**	Can read and write	18	15.1
	Primary	61	51.3
	Secondary	21	17.6
	University	19	16.0
ICD-10 diagnosis*	Bipolar disorder	28	23.5
	Depressive episode	18	15.1
	Recurrent depression	31	26.1
	Dysthymia	42	35.3
**History of psychiatric admissions**	No	89	74.8
	1	19	16.0
	2	3	2.5
	3	2	1.7
	≥4	6	5.0
**No. of Psychiatrists**	1	29	24.4
*Mean 3.4 ± 2.6*	2	31	26.1
*Rank 1-15*	3	19	16.0
*Mode 1; Median 3*	≥4	40	33.5
**Psychotropic drugs**	One drug	19	16.0
*Mean 2.92 ± 1.45 drugs*	Two drugs	29	24.4
*Rank 1-8*	Three drugs	37	31.1
*Polypharmacy %*	Four drugs	16	13.4
	Five or more drugs	18	15.1
**Treatment**	Antidepressants	97	81.5
	Tricyclics	7	5.9
	SSRIs	67	56.3
	SNSRIs	67	56.3
	Benzodiazepines	96	80.7
	Antipsychotics	19	16
	Conventional	2	1.7
	Atypical	18	15.1
	Mood stabilizers	22	18.5
	Anticholinergics	2	1.7
**Self-reported adherence**	Adherent	59	49.6
	Non-adherent	60	50.4

*Abbreviations*: *ICD* International Classification of Diseases, *SNRIs* Selective Noradrenaline Reuptake Inhibitors, *SSRIs* Selective Serotonin Reuptake Inhibitors.

An initial analysis (Pearson correlation analysis) was performed to assess the relationship patterns among the different psychological processes (self-efficacy, psychological reactance, and locus of control), and the depression level (BDI), in order to identify whether there was a high covariability among them or whether they remain independents. Correlation coefficients were also obtained among those processes and the contextual variables usually associated with treatment compliance (age, educational level, treatment duration, and number of different drugs). Finally, using Morisky scale as a continuous variable, level of adherence was correlated with all of those variables.

Table 2 shows the coefficients obtained. From a total of 21 correlation coefficients among psychological processes, only six attained statistical significance. The most prominent was the correlation between the two psychological reactance scales. There was also a considerable positive correlation between cognitive reactance and Chance subscale (locus of control). Despite these coefficients, the general pattern enables identifying the processes as relatively independent by measuring distinctive features.

**Table 2:** **Pearson correlation coefficients (Cramer’s V for sex variable) among the different psychological processes, contextual variables, and the level of adherence/Morisky scale, in a sample of depressive patients (n = 119)**

	**BDI**	**S-E**	**PR-A**	**PR-C**	**HLC-I**	**HLC-Ch**	**HLC-D**	**HLC-O**	**Adherence**
**BDI**		-.61**	.28**	.42**	.01	.35**	-.17*	-.06	.20*
**S-E**			-.04	-.16	.02	-.14	.15	.26^**^	.04
**PR-A**				.55^**^	-.10	.14	-.05	-.00	.32**
**PR-C**					-.09	.36^**^	-.12	-.04	.25**
**HLC-I**						-.03	.18^*^	.14	.01
**HLC-Ch**							.00	.23^*^	.15
**HLC-D**								.17	-.14
**HLC-O**									.12
**Sex(1)**	.58	.40	.50	.33	.48	.57	.48	.48	.18
**Age**	.05	.07	-.01	-.01	-.10	.02	.10	.13	.16
**Educational level**	-.08	.18*	.00	.07	-.04	-.06	.10	-.03	-.06
**Treament duration**	-.07	.10	.05	.01	.02	.10	-.10	.11	.17
**No. different drugs**	.52**	-.27**	.23*	.17*	-.06	.14	-.04	.00	.05

*Abbreviations*: **P < .01; *P < .05; *BDI* Beck Depression Inventory, *PR-A* Psychological Reactance, Affective subscale, *PR-C* Psychological Reactance, Cognitive subscale, *HLC-I* Health Locus of Control, Internal subscale, *HLC-Ch* Health Locus of Control, Chance subscale, *HLC-D*  Health Locus of Control, Doctors subscale, *HLC-O* Health Locus of Control, Others subscale, (1) Cramer’s V correlation.

According to the level of depression, several significant correlations were registered: negative correlations with self-efficacy and with an external locus of control (confidence in their doctors). Positive correlations were obtained for both affective and cognitive psychological reactance variables, chance external locus of control, and level of adherence. Also, a positive correlation was found between the level of depression and higher number of different drugs.

Four statistically significant correlation coefficients with the contextual variables were found. “Number of different drugs” was negatively related to self-efficacy and positively related to psychological reactance (affective and cognitive). Self-efficacy was also positively related to educational level. The remaining coefficients did not attain statistical significance (some of them with correlations close to zero).

Only psychological reactance subscales (affective and cognitive) attained statistical significance with level of adherence, and both in the same direction: as reactance increases there is a lower level of adherence.

A second group of analyses was performed to determine whether the levels in those psychological processes differed between compliant and non-compliant patients (according to categoric Morisky scale classification). Table 3 summarizes the ANOVA obtained (each variable was analyzed independently), including means, standard deviations of locus of control, reactance, and self-efficacy, and a test of normal distribution. As can be observed, the external locus of control variable ‘Doctors’ (the patients’ belief of the importance of their doctors in the outcome of their depressive disorder), is not normally distributed.

**Table 3:** **Means, standard deviations, and ANOVA between adherent and non-adherent depressive outpatients on different psychological processes and the level of depression**

**Variables**	**K-S**	***P***	**Adherent**		**Non-adherent**		**F**	***P***	**Partial η** ^**2**^
			**M**	**SD**	**M**	**SD**			
**HLC –Internal**	.72	.673	3.56	1.11	3.40	1.22	.55	.459	.005
**HLC –Chance**	1.08	.192	3.17	1.12	3.33	1.00	.69	.409	.006
**HLC –Doctors**	1.65	.009	5.05	0.98	4.68	0.96	4.28	.041	.035
**HLC –Others**	.84	.485	3.60	1.28	3.69	1.25	.15	.699	.001
**PR –Affective**	.64	.801	3.04	0.88	3.50	0.72	9.57	.002	.076
**PR –Cognitive**	.91	.379	1.87	0.77	2.24	0.70	7.48	.007	.060
**Self-Efficacy**	.71	.697	25.24	6.81	25.67	6.32	.13	.722	.001
**BDI**	1.15	.144	18.23	11.09	19.15	10.39	.13	.724	.002

*Abbreviations*: *HLC* Health Locus of Control, *PR* Psychological Reactance, *BDI* Beck Depression Inventory, *K-S* Kolmogorov-Smirnov’s z, *M* median, *P* probability, *SD* Standard Deviation.

On examination of the ANOVA results, three statistically significant differences were recorded in the cognitive factors studied; one variable was related to health locus of control and two variables refer to the psychological reactance construct. Adherent patients scored higher on the MHLC Doctors subscale, although this result must be taken with caution because of its non-normal distribution. No other health locus of control variables attained statistical significance. As for psychological reactance, both affective and cognitive subscales attained statistically significant differences between adherent and non-adherent patients: non-adherence patients scored higher for both measures, indicating more of a reactance stance when patients do not follow medical prescriptions.

Taking into account the pattern of correlation coefficients and the differences observed in the last ANOVAs, a logistic regression analysis was performed to ascertain the relative role of these cognitive factors and health cognitions in predicting adherence to prescribed treatment. A step-by-step method was used to select only those variables that play a significant role predicting adherence. Also, to know the stability of the model, a new logistic regression was performed with a random sample (50%). Subsequently, the model obtained was tested with the rest of the sample. Table 4 summarizes the coefficients obtained.

**Table 4:** **Logistic regression predicting compliant and non-compliant depressive patients, according to psychological processes and contextual variables**

**A)Total sample (n = 119)**						
	**B**	**Wald**	***P***	**OR**	**95% CI**	
Age	.07	12.95	.000	1.07	1.03	1.12
HLC-Doctors	-.20	6.42	.011	.82	.71	.96
PR-Affective	.11	9.36	.002	1.12	1.04	1.20
Constant	−3.91	5.40	.020	.020		
**B) Random sample (n = 60)**						
	**B**	***P***	**OR**			
Age	0.07	0.02	1.07			
HLC-Doctors	−0.30	0.02	0.74			
PR-Affective	0.13	0.02	1.14			
Constant	−2.85	0.26	0.06			

*Abbreviations*: *B* beta coefficients, *CI* confidence interval, *HLC* Health Locus of Control, *OR* Odds ratio, *P* probability, *PR* Psychological Reactance.

According to Wald coefficients, only three variables attained statistical significance. Age was the best predictor of adherence to prescribed treatment; older patients were less likely to be adherent. The following variables in the regression analysis refer to psychological processes: a negative association with an external locus of control (there is more compliance when patients rely on their physicians), and a positive relationship with affective reactance, where more reactant patients were less compliant. Variables usually associated with adherence such as treatment complexity (number of different drugs), and treatment duration did not attain statistical significance. This general model attained statistical significance (X^2^(3) = 29.03; p = .000), with a 73% of patients correctly classified (cut-off point .50).

The logistic regression with a random sample (n = 60) attained a similar pattern, with the same three variables into the equation. In this case the percentage of cases correctly classified was 71%. Finally, the projection of this model over the rest of the sample raised a 66% of cases correctly classified.

To ascertain whether there was a specific role for type of medication, Chi-squared analyses were performed for adherence and no-adherence patients and patients who were or were not taking antidepressants, antipsychotics, anxiolytics, or mood stabilizers. No statistically significant differences were obtained (antidepressants: X2[1] = .98; antipsychotics: X2[1] = 1.67; benzodiazepines: X2[1] = .08; mood stabilizers: X2[1] = .01).

## Discussion

The results of this study show statistically significant associations between age, psychological reactance and doctor-attributed health locus of control and adherence to drug treatment in psychiatric outpatients with depression.

Among the socio-demographic factors, age was the variable most consistently related to adherence; older patients were less likely to be adherent. This finding is not in line with that of some previous studies, which found a statistically significant association between older age and a lower probability of (or a longer time to) discontinuation [13]. Although poor adherence to prescribed regimens could affect all age groups the prevalence of cognitive and functional impairments in elderly patients increases their risk of poor adherence in the same way as the presence of multiple co-morbidities, the polypharmacy associated and the subsequent complex medical regimens [38-40]. Moreover, age-related pharmacokinetic and pharmacodynamic abnormalities make this population even more vulnerable to non-adherence by increasing the side effects likely to be experienced from therapy [41,42].

Among the beliefs of having control over one’s health, contrary to some of our hypotheses, not all variables studied influenced the health behavior of adherence since only psychological reactance led to recording a statistically significant association with self-reported adherence. As expected, there was more reactance (especially affective reactance) in less adherent patients. The hypothesis that highly psychological reactant patients would be more likely to be noncompliant than patients showing a low level of psychological reactance was confirmed by our study. Patients with low reactance generally follow instructions and advice, while people with high reactance commonly resist any guidance or assistance. People with high reactance usually have a change style focused on their own resources, personal decisions and initiatives (internal attribution of change), while people with low reactance often seek external help and support to achieve their goals (external attribution change).

The MHLC scales results obtained appear to confirm the idea that depressive patients have appropriate beliefs concerning the management of their condition, and define themselves as informed and aware of the specific ‘commitment rules’ related to depression and, therefore, skilled at exercising due control over it. The higher mean score recorded by depressive patients on the Doctor subscale suggests that depressive patients believe their clinicians play a crucial role in improving their state of health. As for the Other People scale, depressive patients appear to attribute major importance to ‘meaningful others’ in the management of their illness; they believe that interpersonal relationships are fundamentally necessary to manage their condition and, in addition, they have no difficulties placing trust in others. The patients’ high scores on the Internality subscale are indicative, as shown in several previous studies [43], that depressive patients are generally skilled at controlling their pathology and can modify their behavior patterns, in order to improve their state of health. The lower scores on the Chance scale indicate that depressive patients tend to attribute less significance to ‘fate and destiny’ in determining the course of their illness.

However, regarding health locus of control influence on adherence to depression treatment, although internally oriented patients’ self-reported higher adherence to prescribed treatment than externally oriented patients, the differences were not statistically significant. Only the doctors’ subscale was identified as a crucial determinant of adherence with prescribed treatment, with adherent patients scoring significantly higher in believing that psychiatrists were responsible for an improvement in their depression. This general absence of relationship of locus of control with adherence could be mediated by the treatment decision-making. Locus of control is a dimension related to the *causality* of the phenomena: perhaps if patients feel they are a significant part of the treatment outcome, locus of control dimensions would play a relevant role in adherence.

In the case of the self-efficacy process, the absence of differences between adherent and non-adherent patients can be accounted for because it is possible this feature is more related to treatment outcome than treatment process. It is expected that if a patient trusts themself to solve their health problem, they will be more compliant with treatment. However, once again, as we argued for locus of control dimensions, this expectation could be modulated by the treatment decision-making process. Further studies need to be carried out.

Our study has some limitations related to the methodology applied, which need to be recognized. First, although a high rate of participation was recorded in our study, our results could be affected by a selection bias, in that it could be differences in psychological features between those who agree and disagree to participate in our study. Second, our study included a convenient sample of consecutive psychiatric outpatients with depressive disorders who attend community mental health centers and this sample may not be representative of the entire population of depressive patients. Third, all questionnaires applied were self-reports, which could be related to a potential risk of misstatement or could involve response biases. Finally, another limitation of the study is the fact that only one method of estimating adherence was used. At present, several direct and indirect methods for measuring adherence are available. Each method has advantages and disadvantages, and no method is considered the gold standard. Patient questionnaires, i.e. patient self-reports, are the most useful in the clinical setting since they are simple, inexpensive and effectively measure adherence [44-46]. Future studies of the effect of psychological reactance and health locus of control would utilize multiple assessments of adherence (e.g. pharmacy records and self-report). As a result of the limitations reported, some caution is recommended in order to generalize our findings beyond the group studied.

The role shown by our study for psychological reactance in adherence to prescribed treatment for depressed patients would have important implications for clinical practice. Depressed patients with high psychological reactance may perceive a large amount of clinical advice to be a threat to their freedom, and may therefore attempt to reassert this freedom through oppositional or non-compliant behavior [47]. If depressed, reactance prone patients perceive treatment provision to be a threat to their freedom of choice, mental health professionals should be alerted in such a way so as to avoid inducing reactance, and thus non-adherence, to prescribed treatment. Any persuasive message may arouse a motivation to reject the advocacy [48]. Reactant people have a tendency to act without considering potential consequences [49].

In light of the results evidenced in our study it appears important and useful that mental health professionals learn about communication techniques and counseling skills that allow them to avoid the so-called communication barriers: doctors' interventions which can cause non-adherence and hostile attitudes in the patient towards changes [50]. Adequate communication and partnership are not only desirable aims, but they are also possible in the mental health care of depressive patients that implements the basic rights of a group of patients who have not sufficiently benefited from consumer empowerment. Adherence to treatment will benefit from enhancing patients' view that they are responsible for their treatment decisions and when the patients’ viewpoint are taken into account. Nevertheless, this paper is exploratory in nature, and should be read with caution; generalizations should be avoided in the absence of direct empirical evidence of a link between reactance and health behavior. At this time, It would be prudent to hold off making clinical recommendations or trying to generalize the findings to psychiatric patients in general.

## Conclusions

Psychological reactance is an important correlate of adherence to treatment in patients with depressive disorders and this needs to be considered when giving clinical advice in order to avoid inducing reactance and thus non-adherence to prescribed treatments. Mental health professionals need to learn about communication techniques and counseling skills that enable them to deal with the psychological reactance of their patients.
